# Practical Primer Addressing Real-World Use Scenarios of Subcutaneous Vedolizumab in Ulcerative Colitis and Crohn’s Disease: Post Hoc Analyses of VISIBLE Studies

**DOI:** 10.1093/crocol/otad034

**Published:** 2023-08-17

**Authors:** William J Sandborn, Jingjing Chen, Krisztina Kisfalvi, Edward V Loftus, Geert D’Haens, Ninfa Candela, Karen Lasch, Douglas C Wolf, Sharif M Uddin, Silvio Danese

**Affiliations:** Division of Gastroenterology, University of California San Diego, La Jolla, CA, USA; Department of Statistics and Quantitative Sciences, Takeda Development Center Americas Inc., Cambridge, MA, USA; Department of Statistics and Quantitative Sciences, Takeda Development Center Americas Inc., Cambridge, MA, USA; Division of Gastroenterology and Hepatology, Mayo Clinic College of Medicine and Science, Rochester, MN, USA; Department of Gastroenterology, Amsterdam University Medical Centers, Amsterdam, The Netherlands; Department of Gastroenterology, Takeda Pharmaceuticals U.S.A., Inc., Lexington, MA, USA; Department of Gastroenterology, Takeda Pharmaceuticals U.S.A., Inc., Lexington, MA, USA; Department of US Medical, Atlanta Gastroenterology Associates, Atlanta, GA, USA; Department of Gastroenterology, Takeda Pharmaceuticals U.S.A., Inc., Lexington, MA, USA; Gastroenterology and Digestive Endoscopy, IRCCS Ospedale San Raffaele and University Vita-Salute San Raffaele, Milan, Italy

**Keywords:** Crohn’s disease, biologics, inflammation, ulcerative colitis

## Abstract

**Background:**

Vedolizumab, an anti-α_4_β_7_ integrin approved for intravenous (IV) treatment of moderately to severely active ulcerative colitis (UC) and Crohn’s disease (CD), was evaluated as a subcutaneous (SC) formulation in maintenance therapy for UC and CD in phase 3 VISIBLE 1, 2, and open-label extension studies, and recently approved in Europe, Australia, and Canada. Our aim was to evaluate efficacy and safety of IV and SC vedolizumab in clinically relevant UC and CD scenarios.

**Methods:**

Post hoc data analyses from VISIBLE trials examined: (1) whether baseline characteristics predict clinical response to 2 vs 3 IV vedolizumab induction doses; (2) efficacy and safety of switching during maintenance vedolizumab IV to SC in patients with UC; (3) vedolizumab SC after treatment interruption of 1–46 weeks; (4) increasing dose frequency of vedolizumab SC from every 2 weeks (Q2W) to every week (QW) after disease worsening.

**Results:**

No baseline characteristics were identified as strong predictors of response to 2 vs 3 vedolizumab infusions. Most patients achieved clinical response after 2 or 3 doses of IV vedolizumab maintained with SC treatment. Clinical remission and response rates were maintained in patients transitioned from maintenance vedolizumab IV to SC treatment. Of patients with UC, ≥75% achieved response following resumption after dose interruption. Escalation to QW dosing resulted in ≥45% of patients regaining response after loss while receiving vedolizumab Q2W.

**Conclusions:**

Clinical real-world scenarios with vedolizumab SC were reviewed using VISIBLE studies data. Vedolizumab SC provides an additional dosing option for patients with UC and CD.

## Graphical Abstract

**Graphical Abstract:**
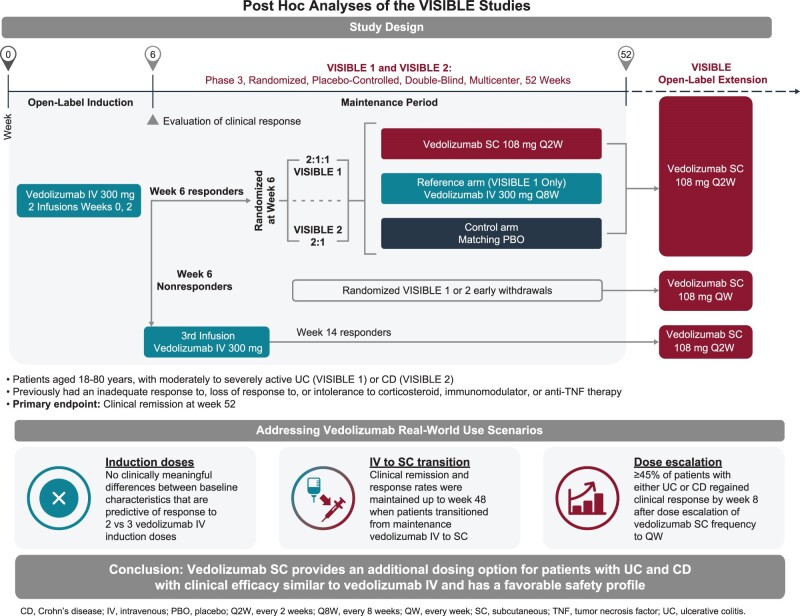

**Key Messages**

- What is already known?
  ◦ Vedolizumab has shown to be effective and safe given subcutaneously (SC) as maintenance therapy after intravenous (IV) induction to treat moderately to severely active ulcerative colitis (UC) and Crohn’s disease (CD).
  ◦ Clinical remission and response rates are maintained in patients transitioning from vedolizumab IV to SC during maintenance therapy.
- What is new here?
  ◦ Clinical baseline characteristics cannot predict which patients require 2 vs 3 IV induction doses to achieve therapeutic response before switching to SC dosing.
  ◦ Consistent with early real-world cohort publications, this randomized trial confirmed that switching from maintenance IV to SC vedolizumab is effective in terms of efficacy and safety.
  ◦ Restarting therapy with SC vedolizumab after interruptions of 1-46 weeks is effective.
  ◦ Escalating the SC vedolizumab dose from once every 2 weeks to once weekly leads to regained response in ≥45% of patients.
- How can this study help patient care?
  - ◦ Reviewing common clinical scenarios likely to be encountered in a real-world setting can allow physicians to make better informed decisions when considering vedolizumab IV and SC dosing in patients with UC or CD.

## Introduction

Vedolizumab is an anti-α_4_β_7_ integrin that selectively blocks lymphocyte trafficking to the gut and is approved as an intravenous (IV) formulation to treat moderately to severely active ulcerative colitis (UC) and Crohn’s disease (CD).^1^ The efficacy and safety of vedolizumab IV 300 mg used in both induction and maintenance therapy are well documented.^2–4^

Many patients with chronic diseases that require long-term maintenance treatment, such as UC and CD, may prefer self-administered subcutaneous (SC) dosing to IV dosing because of the greater convenience and less time-intensive nature of SC therapy.^5–8^ An SC formulation of vedolizumab has been developed as an option for patients who prefer this route of administration. It has been approved for use as maintenance therapy for patients with UC and CD in Argentina, Australia, Canada, and European countries,^1,9,10^ and is undergoing regulatory review in the United States and other countries. In patients transitioning from vedolizumab IV to SC during maintenance therapy, clinical remission and response have been reported to be maintained.^11–15^

The safety and efficacy of maintenance therapy with vedolizumab SC after induction therapy with vedolizumab IV have been evaluated in patients with moderately to severely active UC (VISIBLE 1) and CD (VISIBLE 2), and in a long-term, open-label extension (OLE) of these studies (VISIBLE OLE).^10,16,17^ In VISIBLE 1, significantly higher rates of clinical remission and endoscopic improvement were reported with vedolizumab SC maintenance therapy than with placebo among patients with UC who responded to vedolizumab IV induction.^10^ Similar results for clinical remission were observed in VISIBLE 2 among patients with CD who responded to vedolizumab IV.^16^ The safety and efficacy profiles of vedolizumab SC maintenance therapy were generally similar to those observed with vedolizumab IV.^10,16^ Although VISIBLE OLE is ongoing, interim analyses of this study allow for observation of long-term effects of vedolizumab treatment on safety and efficacy in those patient subgroups with sufficient follow-up to date.

The design of the VISIBLE studies provides an opportunity to examine several common questions encountered in clinical practice about vedolizumab IV and SC dosing in patients with UC and CD. First, are there any factors that predict which patients will respond to 2 doses of vedolizumab IV induction therapy, as per trial design, and which patients may require a third IV dose during the induction phase prior to switching to SC? Second, does transitioning from maintenance vedolizumab IV to SC affect safety and efficacy? Third, what effects on efficacy and safety are seen among initial responders when treatment with vedolizumab SC is initiated after an interruption or “drug holiday” in initial vedolizumab IV treatment? Lastly, does escalating the dosing frequency of vedolizumab SC from every 2 weeks (Q2W) to once weekly (QW) restore response and remission among patients who experience disease worsening or require rescue medication? To answer these questions, we conducted post hoc analyses of results from patients with UC and CD in the VISIBLE 1 and VISIBLE 2 clinical trials and interim analyses of the VISIBLE OLE study.

## Methods

### Study Design

We conducted post hoc analyses of patients with UC or CD who participated in VISIBLE 1 (NCT02611830; EudraCT: 2015-000480-14), VISIBLE 2 (NCT02611817; EudraCT: 2015-000481-58), or VISIBLE OLE (NCT02620046; EudraCT: 2015-000482-31). The designs of these studies have been previously reported.^10,16,17^ Briefly, VISIBLE 1 and 2 were phase 3, randomized, placebo-controlled, double-blind, multicenter, international, 52-week trials evaluating the efficacy and safety of vedolizumab SC as maintenance treatment in adults with moderately to severely active UC (VISIBLE 1) or CD (VISIBLE 2).

Eligible patients included in VISIBLE 1 or 2 were adults aged 18-80 years with moderately to severely active UC or CD who previously had an inadequate response to, loss of response to, or intolerance to corticosteroid, immunomodulator, or anti-tumor necrosis factor (TNF) therapy.^10,16^ In both trials, after a 28-day screening period, patients received open-label vedolizumab 300 mg IV at weeks 0 and 2, and were evaluated for clinical response at week 6 (Figure S1, VISIBLE 1 and VISIBLE 2 study designs). Inclusion and exclusion criteria for VISIBLE 1 and 2 are shown in Table S1, study inclusion and exclusion criteria for VISIBLE 1, and Table S2, study inclusion and exclusion criteria for VISIBLE 2.

In VISIBLE 1, clinical response in UC was defined as a reduction in total Mayo score of ≥3 points and a reduction of ≥30% from baseline with an accompanying decrease in rectal bleeding subscore of ≥1 point or an absolute rectal bleeding subscore of ≤1. Total Mayo score was not available at week 14 of VISIBLE 1 (when patients who had not achieved clinical response after 2 vedolizumab IV infusions at week 6 were evaluated for clinical response to the third vedolizumab IV infusion); therefore, clinical response at this visit was defined as a reduction of ≥2 points and ≥25% from baseline partial Mayo score, with an accompanying decrease in rectal bleeding subscore of ≥1 point or absolute rectal bleeding subscore of ≤1. In VISIBLE 2, clinical response in CD was defined as a ≥70-point decrease in CD Activity Index (CDAI) from baseline. Rank-ordered secondary endpoints included enhanced clinical response, defined as a ≥100-point decline in CDAI score from baseline (week 0) at week 52.

In VISIBLE 1, patients who responded to vedolizumab IV induction at week 6 were then randomized in a 2:1:1 ratio to maintenance treatment with vedolizumab 108 mg SC Q2W (along with placebo IV every 8 weeks [Q8W]), vedolizumab IV 300 mg Q8W (along with placebo SC Q2W), or placebo (SC Q2W along with IV Q8W). In VISIBLE 2, patients who responded to vedolizumab IV induction at week 6 were randomized in a 2:1 ratio to maintenance vedolizumab 108 mg SC or placebo Q2W.

In VISIBLE 1 and 2, patients who did not respond at week 6 (after receiving 2 IV doses of vedolizumab at weeks 0 and 2) received another dose of vedolizumab IV at week 6. Patients who then responded at week 14 were eligible to roll over to the VISIBLE OLE study and receive vedolizumab SC Q2W treatment. The vedolizumab SC dose administered Q2W was selected to provide drug exposure similar to that achieved with vedolizumab 300 mg IV administered Q8W based on average serum vedolizumab concentrations at steady state, calculated using a population pharmacokinetic model that performed dose simulations.^18^ Patients who completed VISIBLE 1 or 2 at week 52 were also eligible to enroll in VISIBLE OLE and receive open-label treatment with vedolizumab SC Q2W. Patients who terminated VISIBLE 1 or 2 early due to disease worsening or need for rescue medications could also enroll in VISIBLE OLE, in which they received vedolizumab SC QW.

Results of an interim analysis of VISIBLE OLE data through May 17, 2019, are presented here. Because the VISIBLE OLE study is ongoing, some patients had not yet completed all study visits at the time of data cut for these analyses. Only evaluable patients for each visit (those who were ongoing in the study and had reached the visit [observed cases]) and those who withdrew from the study before the visit were included in the interim analyses.

### Evaluation of Clinical Remission and Response

For patients with UC in VISIBLE 1, the primary endpoint was the percentage of patients with clinical remission (defined as total Mayo score of ≤2 and no individual subscore of >1) at week 52. Clinical response in VISIBLE 1 was defined as a reduction in total Mayo score of ≥3 and ≥30% from baseline (week 0) (or partial Mayo score of ≥2 and ≥25% from baseline if the total Mayo score was not performed at the visit) with an accompanying decrease in rectal bleeding subscore of ≥1 or absolute rectal bleeding subscore of ≤1. For patients with CD in VISIBLE 2, the primary endpoint was clinical remission (defined as a CDAI score ≤150) at 52 weeks. Clinical response was defined as a ≥70-point decrease in CDAI from baseline.

In the VISIBLE OLE study, clinical response was defined as a reduction in partial Mayo score of ≥2 points and ≥25% from baseline with an accompanying decrease in rectal bleeding score of ≥1 or absolute rectal bleeding subscore of ≤1 in patients with UC, or a decrease in Harvey Bradshaw Index (HBI) score of ≥3 points from baseline in patients with CD. Clinical remission was defined as a partial Mayo score of ≤2 and no individual subscore of >1 for patients with UC, or an HBI score of ≤4 for patients with CD.

Fecal calprotectin was collected as an inflammatory biomarker throughout the VISIBLE studies. Adverse events (AEs), regardless of relationship to study drug, were identified during study visits and from spontaneous reports, and were coded using the Medical Dictionary for Regulatory Activities. Injection-site and hypersensitivity reactions were also evaluated.

### Study Populations and Post Hoc Analyses

To address specific clinical scenarios for the use of vedolizumab IV and SC in both UC and CD, patients were classified for post hoc analyses according to several cohorts as described below.

#### Predictors of response to 2 or 3 IV induction doses in UC and CD patients

The analysis cohort was composed of patients enrolled in both VISIBLE 1 and VISIBLE 2 who received 2 or 3 induction doses of vedolizumab IV. These patients were categorized by whether they received 2 doses or 3 doses and whether they achieved clinical response. Baseline demographic and disease characteristics were evaluated to identify potential predictors of early treatment response. To characterize early response, we analyzed partial Mayo scores, rectal bleeding, stool frequency, and fecal calprotectin for patients with UC, and mean CDAI total scores, daily average liquid or soft stool scores, mean daily abdominal pain scores, and fecal calprotectin concentration for patients with CD.

#### Efficacy and safety of transitioning from IV to SC in patients with UC

All patients in this cohort who had achieved clinical response at week 6 of VISIBLE 1 (after vedolizumab IV induction treatment at weeks 0 and 2) were randomized to receive vedolizumab IV Q8W beginning at week 6 until the end of the 52-week study, and then enrolled in VISIBLE OLE and treated with vedolizumab SC Q2W. Patients received a total of 8 vedolizumab IV infusions. We examined the safety and efficacy of vedolizumab SC treatment in these patients. This analysis could not be performed in patients with CD because there was no maintenance vedolizumab IV arm in VISIBLE 2.

#### Efficacy and safety of vedolizumab SC after IV treatment interruption in UC and CD patients

This post hoc analysis included only patients randomized to placebo in VISIBLE 1 or 2 who either completed those 52-week studies (placebo completers) or terminated early (placebo early terminators) before transitioning to the VISIBLE OLE and treatment with vedolizumab SC. Placebo completers experienced a 46-week interruption in vedolizumab treatment between the end of induction with vedolizumab IV treatment and the initiation of vedolizumab SC Q2W treatment in the VISIBLE OLE study. Placebo early terminators experienced interruptions of 1 to 45 weeks in their vedolizumab treatment, depending on when during VISIBLE 1 or 2 they terminated maintenance treatment and rolled over to vedolizumab SC QW treatment in the VISIBLE OLE study. The initiation of therapy following interruption did not comprise IV induction treatment. In the analyses presented here, placebo early terminators stopped participation in VISIBLE 1 or 2 because of disease worsening or the need for rescue medication. In patients with UC, disease worsening was defined in VISIBLE 1 as a ≥3-point increase from week 6 in partial Mayo score on 2 consecutive visits (or an increase to 9 points on 2 consecutive visits if the week 6 score was >6) and a minimum partial Mayo score of ≥5. In patients with CD, disease worsening was defined in VISIBLE 2 as a ≥100-point increase in CDAI score on 2 consecutive visits from week 6 at any study visit and a minimum CDAI score of 220.

#### Efficacy and safety of dose escalation in UC and CD patients

This analysis cohort was composed of patients who received dose escalation because of treatment failure during the VISIBLE 1 or 2 trials or the OLE study. Patients who experienced treatment failure during VISIBLE 1 or 2 discontinued from those studies and were rolled over to the VISIBLE OLE study where their dose was escalated to vedolizumab SC QW. Patients who experienced treatment failure during vedolizumab SC Q2W treatment in the VISIBLE OLE study (these patients had either completed the VISIBLE 1 or 2 study or had responded in either study at week 14, and had been directly enrolled in VISIBLE OLE) had their dose escalated to vedolizumab SC QW.

Treatment failure was defined as disease worsening, need for rescue medications, or need for surgical intervention. Disease worsening among patients with UC enrolled in VISIBLE 1 or VISIBLE OLE, or among patients with CD enrolled in VISIBLE 2, was evaluated as stated earlier. Disease worsening among patients with CD in the VISIBLE OLE study was defined as a ≥4-point increase in HBI score from the week 0 value on 2 consecutive visits from week 0 (VISIBLE 2) and a minimum HBI score of 7.

### Statistical Analysis

Demographics, baseline disease characteristics, and AEs were evaluated descriptively. The percentages of patients with clinical remission and clinical response (with 95% CIs) were calculated following the definitions used in VISIBLE 1, VISIBLE 2, and VISIBLE OLE. Patients who completed or withdrew from the VISIBLE OLE study with missing data for determination of endpoint status were categorized as nonresponder/nonremitter. Patients ongoing in the VISIBLE OLE study who had missing data for determination of endpoint status were categorized as nonresponder/nonremitter only up to the visit reached by the time of the interim data cut.

### Ethical Considerations

The VISIBLE 1, 2, and OLE studies were conducted in accordance with the Declaration of Helsinki and the International Conference on Harmonisation Guidelines for Good Clinical Practice. All patients provided written informed consent before participation.

## Results

### Patient Disposition

Overall patient disposition results from the VISIBLE 1 and 2 studies have already been published.^10,16^ Patients included in each analysis are shown in Figure S2, patient disposition and patient populations.

### Predictors of Response to 2 or 3 IV Induction Doses in UC and CD Patients

Baseline characteristics of patients who responded after 2 and 3 vedolizumab IV doses are shown in Table 1. No clinically meaningful differences in baseline demographics and disease characteristics were observed between these 2 patient groups (Table 1), with the possible exception of prior failure of anti-TNF treatment. The percentages of patients with moderate vs severe disease were similar across all subgroups. Differences in UC disease location were observed between responders and nonresponders among patients who received 3 IV doses (Table 1).

**Table 1.** Baseline demographics and disease characteristics by clinical response characteristics. Data are mean (SD) unless otherwise specified.

	VISIBLE 1 (UC)				VISIBLE 2 (CD)			
	2 IV doses of vedolizumab (*n* = 383)		3 IV doses of vedolizumab (*n* = 139)^a^		2 IV doses of vedolizumab (*n* = 644)		3 IV doses of vedolizumab (*n* = 176)^b^	
	Responders at week 6 (*n* = 215)	Nonresponders at week 6 (*n* = 168)	Responders at week 14 (*n* = 110)	Nonresponders at week 14 (*n* = 29)	Responders at week 6 (*n* = 412)	Nonresponders at week 6 (*n* = 232)	Responders at week 14 (*n* = 110)	Nonresponders at week 14 (*n* = 66)
Age, y	39.3 (13.1)	42.7 (14.9)	42.8 (14.8)	43.7 (14.8)	37.7 (13.8)	37.0 (12.4)	38.9 (13.6)	36.0 (12.0)
Male, *n* (%)	129 (60.0)	89 (53.0)	61 (55.5)	16 (55.2)	223 (54.1)	116 (50.0)	49 (44.5)	36 (54.5)
Mean BMI, kg/m^2^	24.8 (5.1)	23.7 (4.7)	23.9 (4.7)	24.2 (5.6)	24.6 (5.7)	24.4 (6.2)	24.3 (6.3)	24.8 (6.7)
Duration of UC/CD, y	7.8 (6.4)	7.0 (6.8)	6.7 (6.6)	7.4 (7.6)	9.1 (8.3)	8.7 (7.8)	8.7 (7.6)	8.9 (7.9)
Disease localization in UC, *n* (%)								
Proctosigmoiditis	28 (13.0)	31 (18.5)	18 (16.4)	4 (13.8)	—	—	—	—
Left-sided colitis	93 (43.3)	52 (31.0)	43 (39.1)	5 (17.2)	—	—	—	—
Extensive colitis	16 (7.4)	18 (10.7)	11 (10.0)	4 (13.8)	—	—	—	—
Pancolitis	77 (35.8)	67 (39.9)	38 (34.5)	16 (55.2)	—	—	—	—
Missing	1 (0.5)	0	0	0	—	—	—	—
Disease localization in CD, *n* (%)								
Colon only	—	—	—	—	85 (20.6)	52 (22.4)	27 (24.5)	12 (18.2)
Ileum only	—	—	—	—	89 (21.6)	58 (25.0)	30 (27.3)	19 (28.8)
Ileocolonic	—	—	—	—	193 (46.8)	91 (39.2)	40 (36.4)	26 (39.4)
Other	—	—	—	—	44 (10.7)	31 (13.4)	13 (11.8)	9 (13.6)
Missing	—	—	—	—	1 (0.2)	0	0	0
Prior surgery for CD, n (%)	—	—	—	—	109 (26.5)	72 (31.0)	32 (29.1)	26 (39.4)
Smoking classification, *n* (%)								
Never smoker	139 (64.7)	109 (64.9)	71 (64.5)	16 (55.2)	240 (58.3)	133 (57.3)	65 (59.1)	38 (57.6)
Current smoker	21 (9.8)	7 (4.2)	3 (2.7)	3 (10.3)	80 (19.4)	49 (21.1)	21 (19.1)	13 (19.7)
Ex-smoker	55 (25.6)	52 (31.0)	36 (32.7)	10 (34.5)	92 (22.3)	50 (21.6)	24 (21.8)	15 (22.7)
UC baseline disease activity, *n* (%)								
Total Mayo score								
Mild (Mayo score <6)	0	1 (0.6)	0	1 (3.4)	—	—	—	—
Moderate (Mayo score = 6-8)	85 (39.5)	61 (36.3)	42 (38.2)	12 (41.4)	—	—	—	—
Severe (Mayo score = 9-12)	130 (60.5)	106 (63.1)	68 (61.8)	16 (55.2)	—	—	—	—
Partial Mayo score								
Mayo score < 6	59 (27.4)	49 (29.2)	31 (28.2)	12 (41.4)	—	—	—	—
Mayo score ≥6	156 (72.6)	119 (70.8)	79 (71.8)	17 (58.6)	—	—	—	—
Rectal bleeding	1.7 (0.7)	1.6 (0.8)	1.6 (0.76)	1.4 (0.9)	—	—	—	—
Stool frequency	2.3 (0.8)	2.3 (0.79)	2.4 (0.76)	2.1 (0.98)	—	—	—	—
Mayo endoscopic subscore	2.6 (0.49)	2.7 (0.46)	2.7 (0.44)	2.6 (0.50)	—	—	—	—
History of fistulizing disease in CD, *n* (%)	—	—	—	—	91 (22.1)	56 (24.1)	22 (20.0)	17 (25.8)
Fistula status in CD, *n* (%)								
Draining					28 (6.8)	20 (8.6)	11 (10.0)	4 (6.1)
All closed					19 (4.6)	6 (2.6)	4 (3.6)	
None					365 (88.6)	206 (88.8)	95 (86.4)	62 (93.9)
CD baseline disease activity, *n* (%)								
Moderate (CDAI score ≤300)	—	—	—	—	238 (57.8)	165 (71.1)	78 (70.9)	49 (74.2)
Severe (CDAI score >300)	—	—	—	—	174 (42.2)	67 (28.9)	32 (29.1)	17 (25.8)
CRP (μg/g)	—	—	—	—	16.9 (24.98)	17.9 (25.76)	14.6 (23.60)	13.8 (18.62)
CRP categories (mg/L), *n* (%)								
≤2.87	—	—	—	—	104 (25.2)	65 (28.0)	39 (35.5)	18 (27.3)
>2.87 to ≤5	—	—	—	—	57 (13.8)	30 (12.9)	14 (12.7)	11 (16.7)
>5 to ≤10	—	—	—	—	87 (21.1)	36 (15.5)	16 (14.5)	14 (21.2)
>10	—	—	—	—	164 (39.8)	101 (43.5)	41 (37.3)	23 (34.8)
Fecal calprotectin, µg/g	2608.7 (3354.8)	2984.2 (5015.7)	3253.3 (5870.4)	2451.8 (2185.0)	1344.8 (1782.7)	1852.7 (5362.9)	1865.8 (7360.9)	1231.6 (1425.9)
Fecal calprotectin categories, *n* (%)								
≤250 µg/g	17 (7.9)	12 (7.1)	12 (10.9)	0	79 (19.2)	50 (21.6)	30 (27.3)	15 (22.7)
>250 to ≤500 µg/g	18 (8.4)	16 (9.5)	10 (9.1)	3 (10.3)	70 (17.0)	41 (17.7)	22 (20.0)	11 (16.7)
>500 µg/g	175 (81.4)	137 (81.5)	87 (79.1)	26 (89.7)	259 (62.9)	140 (60.3)	58 (52.7)	39 (59.1)
Missing	5 (2.3)	3 (1.8)	1 (0.9)	0	4 (1.0)	1 (0.4)	0	1 (1.5)
Prior anti-TNF use, *n* (%)	84 (39.1)	83 (49.4)	52 (47.3)	18 (62.1)	240 (58.3)	136 (58.6)	60 (54.5)	42 (63.6)
Prior anti-TNF failure, *n* (%)^c^	84 (39.1)	75 (44.6)	49 (44.5)	16 (55.2)	208 (50.5)	125 (53.9)	54 (49.1)	38 (57.6)
Concomitant oral corticosteroid use, *n* (%)	91 (42.3)	66 (39.3)	50 (45.5)	15 (51.7)	140 (34.0)	82 (35.3)	40 (36.4)	18 (27.3)
Concomitant oral immunomodulator use^a^*n* (%)	72 (33.5)	70 (41.7)	47 (42.7)	13 (44.8)	131 (31.8)	63 (27.2)	34 (30.9)	16 (24.2)
Albumin, g/L	42.7 (3.64)	41.8 (4.47)	41.6 (4.41)	42.4 (4.47)	41.9 (4.60)	41.1 (5.38)	41.6 (5.48)	41.1 (5.14)
Hemoglobin, g/L	127.8 (17.7)	123.5 (18.7)	122.6 (19.1)	127.0 (18.0)	130.1 (16.5)	127.2 (17.6)	128.5 (17.7)	126.7 (17.0)

*Notes*: For patients with UC, clinical remission was defined as total Mayo score of ≤2 and no individual subscore >1 at week 52, and clinical response was defined as a reduction in total Mayo score of ≥3 and ≥30% from baseline (week 0) (or partial Mayo score of ≥2 and ≥25% from baseline if the total Mayo score was not performed at the visit) with an accompanying decrease in rectal bleeding subscore of ≥1 or absolute rectal bleeding subscore of ≤1. For patients with CD, clinical remission was defined as a CDAI score ≤150 at 52 weeks, and clinical response was defined as a ≥70-point decrease in CDAI score from baseline.

^a^Four patients received a third IV dose although they achieved clinical response at week 6. Those patients are excluded from this group.

^b^Sixteen patients received a third IV dose although they achieved clinical response at week 6. Those patients are excluded from this group.

^c^Based on total patients, not based on patients exposed to anti-TNF-α.

Abbreviations: BMI, body mass index; CD, Crohn’s disease; CDAI, Crohn’s Disease Activity Index; CRP, C-reactive protein; IV, intravenous; TNF, tumor necrosis factor; UC, ulcerative colitis.

**Figure 1.**
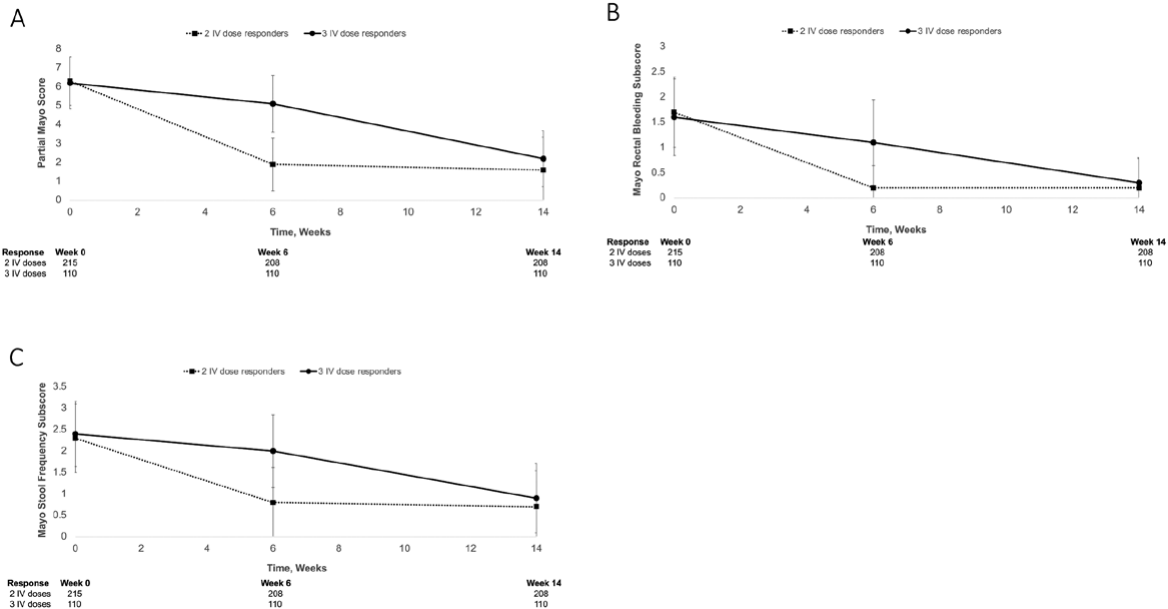
Changes in disease characteristics among patients with ulcerative colitis in VISIBLE 1 receiving 2 or 3 induction doses of vedolizumab IV. (A) Partial Mayo score; (B) rectal bleeding subscore; (C) Mayo stool frequency subscore. IV, intravenous.

**Figure 2.**
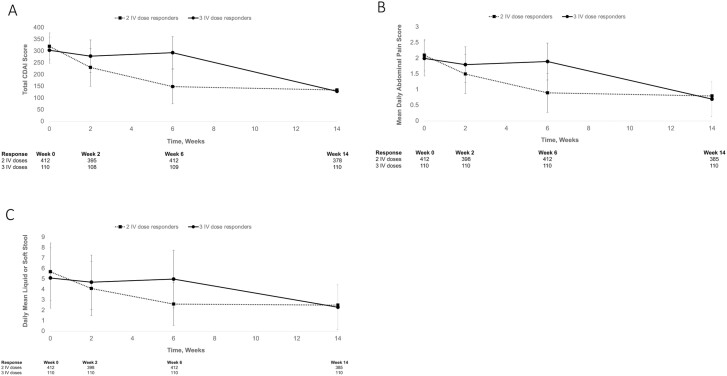
Changes in disease characteristics among patients with Crohn’s disease in VISIBLE 2 receiving 2 or 3 induction doses of vedolizumab IV. (A) Crohn’s Disease Activity Index total score; (B) daily abdominal pain score; (C) daily liquid/soft stools. CDAI, Crohn’s Disease Activity Index; IV, intravenous.

A higher percentage of nonresponders had extensive colitis, especially at week 14. Partial Mayo scores in UC (Figure 1A), mean CDAI total scores (Figure 2A), mean daily abdominal pain scores in CD (Figure 2B), and daily average liquid or soft stool scores (Figure 2C) were similar in both groups of responders at baseline, and were substantially different at week 6 before becoming similar again at week 14, suggesting there may not be long-term differences between the 2 groups of responders. Similar patterns were observed for rectal bleeding and stool frequency in UC (Figures 1B and 1C). Mean (SD) fecal calprotectin concentrations at both baseline and week 6 were lower among patients who responded after 2 IV doses (2608.7 [3354.8] µg/g at baseline; 1821.2 [6809.8] µg/g at week 6) compared with patients who responded after 3 IV doses (3253.3 [5870.4] µg/g at baseline; 3449.5 [7839.2] at week 6).

### Efficacy and Safety of Transitioning From Vedolizumab IV to SC in UC Patients

**Figure 3.**
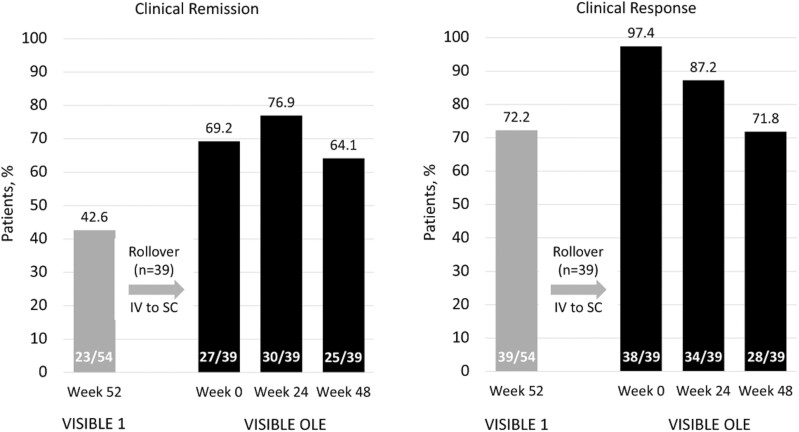
Clinical response and remission rates before and after transitioning from vedolizumab IV to SC after 8 IV infusions. IV, intravenous; OLE, open-label extension; SC, subcutaneous.

Thirty-nine patients completed 8 vedolizumab IV infusions over 52 weeks in VISIBLE 1 and received SC doses in the VISIBLE OLE study. Among these patients, 59% were male, the mean age was 41 years, and the duration of UC was 9.1 years (Table S3, demographics and baseline characteristics for patients with UC who transitioned from IV to SC). At baseline for VISIBLE 1, one-third of these patients had moderately active UC as assessed by Mayo score, and two-thirds had severely active UC (Mayo score 9-12). Clinical remission and clinical response rates were maintained during vedolizumab SC treatment through week 48 (clinical remission: 64.1%; clinical response: 71.8%; Figure 3). Treatment-emergent AEs (TEAEs) following the transition to vedolizumab SC were mostly mild. Five serious TEAEs occurred, and 1 patient experienced 2 TEAEs that led to discontinuation of study drug (grade 3B follicular lymphoma and diffuse large B-cell lymphoma; Table 2).

**Table 2.** Adverse events among patients who transitioned (IV to SC) after 8 vedolizumab IV doses in VISIBLE 1.

	No. of events	Patients (%) (*n* = 39)
Any AE	62	22 (56.4)
Related	6	4 (10.3)
Not related	56	18 (46.2)
Mild	46	12 (30.8)
Moderate	14	8 (20.5)
Severe	2	2 (5.1)
Serious AE	5	5 (12.8)
Not related	5	5 (12.8)
AEs leading to study drug discontinuation	2	1 (2.6)
Injection-site reactions	3	3 (7.7)
Hypersensitivity reactions	2	2 (5.1)

Abbreviations: AE, adverse event; IV, intravenous; SC, subcutaneous.

### Efficacy and Safety After Treatment Interruption

#### UC efficacy

Of the 56 patients randomized to placebo in the VISIBLE 1 trial, 20 (36%) completed the week 52 visit and had received placebo for 46 weeks. All these patients were enrolled in VISIBLE OLE (placebo completers). Of the 36 patients receiving placebo who terminated early from VISIBLE 1, 32 enrolled in VISIBLE OLE (placebo early terminators). Baseline demographics of patients with UC who experienced treatment interruption are shown in Table S4, demographics and baseline characteristics for patients with UC who experienced vedolizumab treatment interruptions in VISIBLE 1.

**Figure 4.**
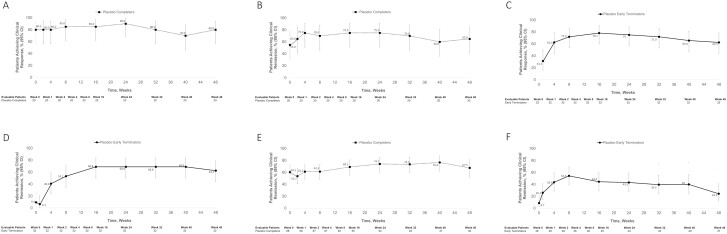
Clinical response and remission following vedolizumab treatment interruption. (A) Clinical response in UC, placebo completers; (B) clinical remission in UC, placebo completers; (C) clinical response in UC, placebo early terminators; (D) clinical remission in UC, placebo early terminators; (E) clinical remission in CD, placebo completers; (F) clinical remission in CD, placebo early terminators. Placebo completers = responders to 2 doses of vedolizumab IV induction before receiving placebo from week 14 to week 52. Placebo early terminators = patients who received 0-30 weeks of placebo and had 2 vedolizumab IV induction doses. CD, Crohn’s disease; IV, intravenous; UC, ulcerative colitis.

Among patients with UC who responded to vedolizumab IV induction and received placebo for 46 weeks in VISIBLE 1, 16 patients (80%) displayed clinical response at week 0 of VISIBLE OLE, 18 patients (90%) at week 24, and 16 patients (80%) at week 48 after reinitiation of vedolizumab treatment with vedolizumab SC (Figure 4A). Among placebo completers, 11 patients (55.0%) displayed clinical remission at week 0 of VISIBLE OLE, increasing to 15 patients (75.0%) at week 24, and 13 patients (65.0%) at week 48 after reinitiation of vedolizumab treatment with vedolizumab SC (Figure 4B). Among patients who responded to vedolizumab IV induction, were randomized to placebo, and terminated early from VISIBLE 1 (placebo early terminators), a larger increase in clinical response was observed from week 1 of VISIBLE OLE (10 patients; 31.3%) to week 24 (24 patients; 75.0%) and week 48 (20 patients; 62.5%; (Figure 4C). Of the evaluable early terminators, 3 patients (9.4%) were in clinical remission at week 0 of VISIBLE OLE (Figure 4D). In addition, 22 patients (68.8%) and 20 patients (62.5%) achieved clinical remission at weeks 24 and 48, respectively (Figure 4D).

#### CD efficacy

Of the 135 patients randomized to placebo in VISIBLE 2, 68 completed the week 52 visit, and had received placebo for 46 weeks. All of these patients were enrolled in the VISIBLE OLE study (placebo completers). Among the 61 patients receiving placebo who terminated early from VISIBLE 2, 46 enrolled in VISIBLE OLE (placebo early terminators). Baseline demographics of patients with CD who experienced treatment interruption are shown in Table S5, demographics and baseline characteristics for patients with CD who experienced vedolizumab dose interruptions in VISIBLE 2. Among patients with CD who responded to vedolizumab induction, received placebo, and then reinitiated vedolizumab SC in VISIBLE OLE, 41 patients (60.3%) at week 0, 40 patients (74.1%) at week 24, and 27 patients (67.5%) at week 48 displayed clinical remission (Figure 4E). Among early terminators with CD, 16 patients (34.8%) at week 1, 19 patients (43.2%) at week 24, and 13 patients (35.1%) at week 48 achieved clinical response. Among early terminators with CD, 4 patients (8.7%) at week 0, 19 patients (43.2%) at week 24, and 9 patients (24.3%) at week 48 displayed clinical remission (Figure 4F).

#### Safety in UC and CD

No new safety signals were observed in this treatment interruption cohort (Table S6, AEs during vedolizumab treatment for UC following treatment interruption in VISIBLE 1, and Table S7, AEs during vedolizumab treatment for CD following treatment interruption in VISIBLE 2). More AEs, including injection-site and hypersensitivity reactions, were observed in placebo early terminators than in placebo completers. Two patients with UC experienced AEs leading to study drug discontinuation (UC flare and worsening of UC). Four patients with CD experienced 5 AEs leading to study drug discontinuation: worsening of CD, arthralgia in large joints (ankles, knees, elbows, and wrists), increased amylase, increased lipase, and CD exacerbation. Among all patients experiencing treatment interruption, 6 (11.5%) with UC and 19 (16.7%) with CD experienced serious TEAEs. Only 1 serious AE of anal fistula in a patient with CD was considered to be related to study treatment.

### Efficacy and Safety of Dose Escalation

#### UC efficacy

**Figure 5.**
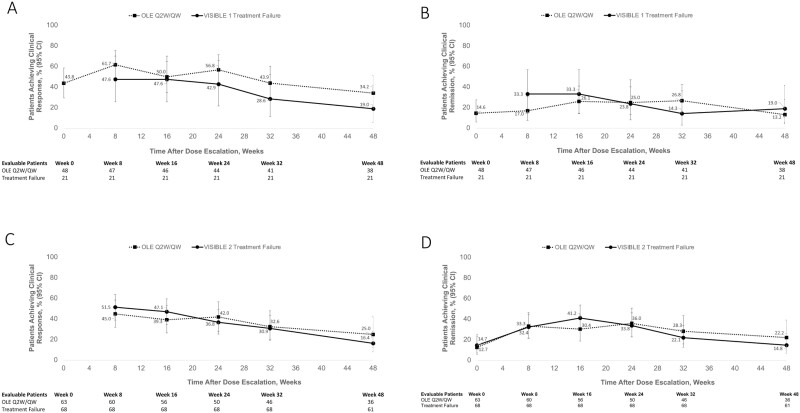
Clinical response and remission rates among patients who experienced vedolizumab dose escalation. OLE Q2W/QW = patients for whom Q2W SC failed during the OLE after at least 1 dose before transitioning to QW SC. VISIBLE 1 or 2 failures = patients who have had less than 38 weeks on Q2W SC in VISIBLE 1 or 2 before transitioning to QW SC immediately on entry to OLE. Available data are shown; data were collected at different time points for different groups. CD, Crohn’s disease; OLE, open-label extension; QW, every week; Q2W, every 2 weeks; UC, ulcerative colitis.

Of the 383 patients with UC who enrolled in VISIBLE 1, 49 patients rolled over to the VISIBLE OLE study with Q2W dosing but later required dose escalation (week 14 responders and VISIBLE 1 completers [OLE Q2W/QW cohort]). A total of 21 patients were randomized to the vedolizumab SC arm (Q2W) in VISIBLE 1 but terminated early because of treatment failure; these patients received vedolizumab SC QW when they entered the VISIBLE OLE study (VISIBLE 1 treatment failure cohort). Of the 70 patients with UC who experienced dose escalation, 75.7% had severe disease activity (Mayo score 9-12) at VISIBLE 1 baseline, and mean patient age was 39 years, with a 7.3-year duration of UC (Table S8, demographics and baseline characteristics for patients with UC who received vedolizumab dose escalations). Among evaluable patients with UC who received vedolizumab dose escalation during the VISIBLE OLE study or after treatment failure in VISIBLE 1, 29 patients (61.7%) and 10 patients (47.6%), respectively, achieved clinical response by week 8 following dose escalation, and 8 patients (17%) and 7 patients (33.3%) recaptured clinical remission. At 48 weeks after the initiation of dose escalation, 13 patients (34.2%) and 4 patients (19.0%) had clinical response, and 5 patients (13.2%) and 4 patients (19.0%) were in clinical remission (Figures 5A and 5B).

#### CD efficacy

Of the 410 patients with CD who enrolled in the VISIBLE 2 study, 63 rolled over to the VISIBLE OLE study with Q2W dosing but later required dose escalation (VISIBLE OLE Q2W/QW cohort). A total of 68 patients were randomized to the vedolizumab SC arm (Q2W) but terminated early because of treatment failure and received vedolizumab SC QW when they entered VISIBLE OLE (VISIBLE 2 treatment failure cohort). Of the 131 patients with CD who experienced dose escalation, 44% had severe disease activity (CDAI scores >300); mean CD duration was 9.3 years (Table S9, demographics and baseline characteristics for patients with CD who received vedolizumab dose escalations).

Of evaluable patients with CD who received vedolizumab dose escalation during VISIBLE OLE or after treatment failure in VISIBLE 2, clinical response was achieved in 27 patients (45.0%) and 35 patients (51.5%) at 8 weeks after dose escalation, respectively, and 9 patients (25.0%) and 10 patients (16.4%) at 48 weeks after the initiation of dose escalation, respectively (Figure 5C). Clinical remission was achieved by 20 patients (33.3%) and 22 patients (32.4%) at week 8 and by 8 patients (22.2%) and 9 patients (14.8%) at week 48 (Figure 5D). Due to the interim nature of these analyses, not all patients had reached week 48 by the time these analyses were conducted.

#### Safety in UC and CD

The majority of TEAEs were mild or moderate (Table S10, AEs following vedolizumab dose escalation in patients with UC, and Table S11, AEs following vedolizumab dose escalation in patients with CD). A total of 6 patients with UC experienced TEAEs leading to discontinuation of study drug (UC flare, cytomegalovirus infection of intestine, no improvement of colitis ulcerosa, osteoporosis, worsening of UC, and anemia). Four patients with CD experienced TEAEs leading to discontinuation of study drug (worsening of CD [2 instances], exacerbation of CD, and headache). Three serious AEs (2 events of muscular weakness and myalgia in 1 patient and 1 event of colitis ulcerative in 1 patient) among patients with UC and 3 serious AEs (1 event of tubulointerstitial nephritis in 1 patient, *Clostridium difficile* infection and CD in 2 patients) among patients with CD were considered related to treatment.

## Discussion

The results of these analyses provide insight into the real-world questions that physicians may have about dosing vedolizumab IV and SC. Clinical response was achieved by more than 60% of patients with UC or CD after either 2 or 3 open-label vedolizumab IV infusions. Post hoc analyses revealed no clinically meaningful differences in baseline characteristics that could serve as predictors of response to 2 vs 3 vedolizumab IV doses, with the possible exception of prior failure of anti-TNF treatment. Patients with extensive colitis were less likely to respond even after 3 doses, possibly because the extent of the disease may affect drug pharmacokinetics. Studies have reported conflicting findings about the ability of such factors as disease location, disease severity, high C-reactive protein (CRP) levels, prior surgery, and prior response to anti-TNF therapy to predict treatment response.^4,19–25^ In a study evaluating factors associated with corticosteroid-free remission (defined as a full Mayo score of ≤2, no subscore >1) during vedolizumab therapy, analyses found that absence of exposure to a TNF antagonist, disease duration of ≥2 years, baseline endoscopic activity, and baseline albumin concentration were independently associated with corticosteroid-free remission.^26^ Furthermore, a clinical decision support tool (CDST) has been developed to predict the probability of response to vedolizumab in patients with CD. Predictors of response included when using the CDST are (1) prior bowel surgery; (2) anti-TNF exposure; (3) prior fistulizing disease; (4) baseline albumin levels; and (5) baseline CRP levels.^27^ However, additional evidence from real-world clinical experience or research may help further elucidate potential predictors of response to vedolizumab.

While a deliberate new start of vedolizumab therapy in real-world clinical practice would likely include IV reinduction, reported clinical trial data were based on patient flow per trial design. This did not include a reinduction given that it would have broken the blind from the 52-week portion of the trial, as patients rolled over to OLE SC. Therefore, with caveats, these data are valuable to understand unexpected or unanticipated drug holidays should they occur within the context of current clinical practice/discretion on reinduction.

In patients with UC who experienced a clinical response to induction therapy with vedolizumab IV, therapeutic benefit was maintained from week 52 in VISIBLE 1 following a transition to vedolizumab SC maintenance treatment at week 0 of VISIBLE OLE. Moreover, the safety and tolerability profile of vedolizumab was maintained after the transition from IV to SC. These results may be even more important now, as patients and healthcare providers may be considering transitioning to SC injections from IV infusions in regions where they are available to avoid the need to go to infusion centers or clinics during a pandemic, such as COVID-19. Versatile dosing options may allow physicians and adult patients to choose the delivery method that works best for them.

Due to the nature of UC and CD, most patients require lifelong treatment; therefore, episodic use of biologics is not recommended. Nevertheless, unplanned circumstances can result in long- or short-term treatment interruptions in real-world situations. Thus, understanding the effects of these interruptions can be useful to clinicians. Our analysis confirmed that clinical benefits of vedolizumab treatment were regained in patients who responded to initial vedolizumab IV induction therapy, experienced treatment interruptions of 1-46 weeks, and then reinitiated treatment with vedolizumab SC. These findings were also observed in the GEMINI long-term safety (LTS) study of vedolizumab IV in patients with UC in which they reinitiated vedolizumab after up to 1 year of interrupted therapy.^28^ In this study, retreatment with vedolizumab was as efficacious in achieving clinical response and remission as was initial vedolizumab induction therapy in the same population. At week 8 of retreatment in GEMINI LTS, clinical remission was observed in 73% of patients compared with 69% of patients at week 6 in the GEMINI 1 study. Further, GEMINI LTS also demonstrated the long-term safety of vedolizumab, with a low incidence of both infusion-site reactions and serious treatment-related AEs. Safety and efficacy of reinitiation of vedolizumab treatment after a dose interruption are likely possible because of the low immunogenicity of vedolizumab. In particular, the prevalence of antidrug antibodies is substantially higher in patients retreated with vedolizumab compared with patients receiving continuous treatment.^29^ However, these rates decrease during retreatment with vedolizumab and no relationship has been observed between immunogenicity and infusion-related reactions.^29^

In a recent study of treatment patterns among patients with moderate-to-severe UC in the United States and Europe, clinicians indicated that 13%-39% of patients prescribed biologic therapy (including adalimumab, infliximab, and vedolizumab) had received an escalated dose (ie, a higher-than-indicated dose or greater-than-indicated dosing frequency).^30^ A systematic review with meta-analysis demonstrated that approximately one-third of patients with CD experienced a loss of response and required dose intensification in primary anti-TNF-α responders.^31^ Although most patients with UC enrolled in the VISIBLE clinical trials who received vedolizumab SC Q2W dosing did not require dose escalation, previous studies have demonstrated that dose escalation may provide benefit in some patients. A single-institution retrospective analysis of patients with UC who received vedolizumab found that 52 out of 90 patients achieved and maintained remission on standard dosing.^32^ Twenty-four patients received dose escalation (19 partial responders, 2 nonresponders, and 3 patients who relapsed after achieving remission); 10 patients achieved corticosteroid-free remission, and 10 patients experienced clinical improvement. The 2 nonresponders showed no improvement. Patients who had previously received anti-TNF therapy or who had more severe disease at baseline were more likely to require dose escalation.

Our post hoc analysis also demonstrated that some patients who lost response during treatment with vedolizumab SC Q2W achieved clinical response once the frequency of their vedolizumab SC dose was escalated to QW. AEs experienced by patients who received dose escalation were primarily deemed to be unrelated to treatment. Because the VISIBLE OLE study is ongoing and the data presented are interim results, further follow-up of this analysis may provide more insight into the long-term efficacy and safety of dose escalation with vedolizumab SC.

The statistical limitations of post hoc analyses, such as this study, should be noted. The relatively small sample size of the subgroup analyses for treatment interruption, dose escalation, and IV to SC transition are considerations. In addition, although our analysis did not identify any predictors of response to 2 or 3 doses of IV vedolizumab, we only assessed a descriptive analysis of those patients who were categorized as responders and nonresponders. Another limitation is the absence of endoscopic data in the CD population.

## Conclusions

This post hoc analysis demonstrated that clinical response was achieved by most patients with UC or CD after either 2 or 3 doses of vedolizumab IV. Clinical remission and clinical response rates were maintained for UC and CD patients transitioned from maintenance IV to vedolizumab SC treatment. For a majority of patients with UC who experienced a treatment interruption, response was achieved with vedolizumab SC treatment. In patients with UC or CD who lost their response on vedolizumab SC Q2W dosing, dose escalation led to some patients regaining response, but longer term follow-up data are required to assess the true benefit. We reviewed common clinical scenarios likely to be encountered in real-world practice with vedolizumab SC using relevant data from the VISIBLE development program. Overall, vedolizumab SC provided an additional dosing option for patients with UC and CD, with clinical efficacy similar to vedolizumab IV and a favorable safety profile.

## Supplementary Material

otad034_suppl_Supplementary_Material

## Data Availability

The data that support the findings of this study are available from the corresponding author upon reasonable request.
