# Correction to: Practical Primer Addressing Real-World Use Scenarios of Subcutaneous Vedolizumab in Ulcerative Colitis and Crohn’s Disease: Post Hoc Analyses of VISIBLE Studies

**DOI:** 10.1093/crocol/otad071

**Published:** 2023-11-24

This is a correction to: William J Sandborn, Jingjing Chen, Krisztina Kisfalvi, Edward V Loftus, Geert D’Haens, Ninfa Candela, Karen Lasch, Douglas C Wolf, Sharif M Uddin, Silvio Danese, Practical Primer Addressing Real-World Use Scenarios of Subcutaneous Vedolizumab in Ulcerative Colitis and Crohn’s Disease: Post Hoc Analyses of VISIBLE Studies, *Crohn’s & Colitis 360*, Volume 5, Issue 3, July 2023, otad034, https://doi.org/10.1093/crocol/otad034

In the originally published version of this manuscript there was an error in the definition of clinical remission in the second paragraph underneath the heading “Evaluation of Clinical Remission and Response”. The sentence incorrect read:

“Clinical remission was defined as a partial Mayo score of ≥2 and no individual subscore of >1 for patients with UC, or an HBI score of ≤4 for patients with CD.”

This has now been corrected to:

“Clinical remission was defined as a partial Mayo score of ≤2 and no individual subscore of >1 for patients with UC, or an HBI score of ≤4 for patients with CD.”

This error has been corrected.

