# Supplementary material for: Practical Primer Addressing Real-World Use Scenarios of Subcutaneous Vedolizumab in Ulcerative Colitis and Crohn’s Disease: Post Hoc Analyses of VISIBLE Studies
Source: Crohns Colitis 360. 2023 Aug 17;5(3):otad034. doi: 10.1093/crocol/otad034 (PMC10449415; doi:10.1093/crocol/otad034)
Supplement: otad034_suppl_Supplementary_Material [file otad034_suppl_supplementary_material.pdf]

SUPPLEMENTARY MATERIAL FOR:

Practical Primer Addressing Real-World Use Scenarios of Subcutaneous Vedolizumab in Ulcerative Colitis and Crohn's Disease: Post Hoc Analyses of VISIBLE Studies

TABLE S1. Study Inclusion and Exclusion Criteria for VISIBLE 1[1]

**Inclusion**
• Adults aged 18-80 years<br>• Diagnosis of ulcerative colitis (UC) established ≥6 months before screening by clinical and endoscopic evidence and corroborated by a histopathology report<br>• Moderately-to-severely active UC as determined by a total Mayo score of 6 to 12 (with an endoscopic subscore of ≥2) within 10 days before the first dose of study drug. The endoscopy could be performed during the screening period (day –10 to day –5 to allow for central reading prior to first dose at week 0)<br>• Evidence of UC extending proximal to the rectum (≥15 cm of involved colon)<br>• Patients with >8 years' duration of extensive colitis or pancolitis, or left-sided colitis with >12 years' duration; must have documented surveillance endoscopy performed within 12 months of screening<br>• Cancer surveillance must be up-to-date in patients with known risk factors, or be conducted during screening<br>• Inadequate response to, loss of response to, or intolerance of ≥1 of the following: immunomodulators, corticosteroids, or anti-tumor necrosis factor therapies
**Exclusion**
Gastrointestinal exclusion criteria<br>• Abdominal abscess, extensive colonic resection, subtotal or total colectomy<br>• History of >3 small bowel resections or diagnosis of short bowel syndrome<br>• Receipt of tube feeding, defined formula diets, or parenteral alimentation within 28 days before administration of the first dose of study drug<br>• Previous ileostomy, colostomy, or known fixed symptomatic stenosis of the intestine<br>• Receipt of any investigational or approved biologic/biosimilar within 60 days or 5 half-lives of screening, or receipt of any nonpermitted investigational or approved nonbiologic therapies within 30 days or 5 half-lives of screening<br>– Oral 5-aminosalicylic acid (ASA) probiotics and antibiotics were permitted if doses were stable for 2 weeks prior to the first dose of the study and remained stable throughout the study. Antidiarrheals were permitted. Azathioprine, 6-mercaptopurine, or methotrexate could be continued if the patient's dose had been stable for 8 weeks prior to the start of the study<br>• Topical (rectal) treatment with 5-ASAs or corticosteroid enemas/suppositories within 2 weeks of the administration of the first dose of study drug

- Requirement or anticipated requirement for surgical intervention for UC during the study
- History or evidence of adenomatous colonic polyps that had not been removed, or colonic mucosal dysplasia
- Suspected or confirmed diagnosis of ulcerative colitis, indeterminate colitis, ischemic colitis, radiation colitis, diverticular disease associated with colitis, or microscopic colitis

Infectious disease exclusion criteria

- Evidence of an active infection during the screening period
- Evidence of, or treatment for, *Clostridium difficile* infection or other intestinal pathogen within 28 days before the first dose of study drug
- Chronic hepatitis B virus (HBV) infection or chronic hepatitis C virus infection
    - Patients with HBV immunity may have been included
- Active or latent tuberculosis
- Any identified congenital or acquired immunodeficiency (eg, common variable immunodeficiency, HIV infection, organ transplantation)
- Receipt of any live vaccinations within 30 days prior to screening
- Clinically significant infection (eg, pneumonia, pyelonephritis) within 30 days prior to screening, or ongoing chronic infection
- Use of a topical treatment with 5-ASAs or corticosteroid enemas/suppositories within 2 weeks of the first dose of study drug

General exclusion criteria

- Previous exposure to approved or investigational anti-integrin antibodies (eg, natalizumab, efalizumab, etrolizumab, AMG 181), antimucosal addressin cell adhesion molecule-1 (MAdCAM-1) antibodies, or rituximab
- Previous exposure to vedolizumab
- Hypersensitivity or allergies to any vedolizumab excipients
- Any unstable or uncontrolled cardiovascular, pulmonary, hepatic, renal, gastrointestinal, genitourinary, hematological, coagulation, immunological, endocrine/metabolic, or other medical disorder that, in the opinion of the investigator, would confound the study results or compromise patient safety
- Any surgical procedure requiring general anesthesia within 30 days prior to screening or plans to undergo major surgery during the study period
- Any history of malignancy, except for the following: (a) adequately treated nonmetastatic basal cell skin cancer, (b) squamous cell skin cancer that had been adequately treated and that had not recurred for at least 1 year before screening, and (c) history of cervical carcinoma in situ that had been adequately treated and that had not recurred for at least 3 years before screening. Patients with remote history of malignancy (eg, >10 years since completion of curative therapy without recurrence) were to be considered on a case-by-case basis based on the nature of the malignancy and the therapy received
- History of any major neurological disorders, including stroke, multiple sclerosis, brain tumor, or neurodegenerative disease

- Positive progressive multifocal leukoencephalopathy subjective symptom checklist at screening (or before administration of the first dose of study drug at week 0)
- Any of the following laboratory abnormalities during the screening period:
  - Hemoglobin level <8 g/dL
  - White blood cell count <3 × 10^9/L
  - Lymphocyte count <0.5 × 10^9/L
  - Platelet count <100 × 10^9/L or >1200 × 10^9/L
  - Alanine aminotransferase or aspartate aminotransferase >3 × upper limit of normal (ULN)
  - Alkaline phosphatase >3 × ULN
  - Serum creatinine >2 × ULN
- History of drug abuse (defined as any illicit drug use) or a history of alcohol abuse within 1 year before screening
- Active psychiatric problem that, in the investigator's opinion, may have interfered with compliance with study procedures
- Inability of patient or caregiver to attend all the study visits or comply with study procedures
- Unwillingness or inability to self-inject, or lack of a caregiver (defined as a legal adult) to inject the study medication
- Lactation or pregnancy during the screening period or a positive urine pregnancy test at week 0 before study drug administration
- Intention to become pregnant before, during, or within 18 weeks after participating in this study
- Immediate family member, study site employee, or involvement in a dependent relationship with a study site employee who was involved in conduct of this study (eg, spouse, parent, child, sibling), or may have consented under duress

TABLE S2. Study Inclusion and Exclusion Criteria for VISIBLE 2[2]

Inclusion
• Adults aged 18-80 years<br>• Diagnosis of Crohn's disease (CD) established ≥3 months before screening by clinical and endoscopic evidence and corroborated by a histopathology report. Cases of CD established at least 6 months prior to screening for which a histopathology report was not available were to be considered on a case-by-case basis based on the weight of evidence supporting the diagnosis and excluding other potential diagnoses<br>• Moderately-to-severely active CD (Crohn's Disease Activity Index score of 220–450) within 7 days prior to the first dose of study drug and one of the following:<br>– C-reactive protein >2.87 mg/L during the screening period<br>– Ileocolonoscopy with a minimum of 3 nonanastomotic ulcerations (each >0.5 cm in diameter) or 10 aphthous ulcerations (involving a minimum of 10 contiguous cm of intestine) consistent with CD within 4 months before screening<br>– Fecal calprotectin >250 µg/g of stool during the screening period in conjunction with computed tomography enterography, magnetic resonance enterography, contrast-enhanced small bowel radiography, or wireless capsule endoscopy revealing CD ulcerations (aphthae not sufficient) within 4 months before screening<br>• Involvement of the ileum and/or colon, at a minimum<br>• More than 8 years' duration of extensive colitis or pancolitis, or left-sided colitis of >12 years' duration with documented surveillance endoscopy performed within 12 months of screening<br>• Cancer surveillance must be up-to-date in patients with known risk factors, or conducted during screening<br>• Inadequate response to, loss of response to, or intolerance of ≥1 of the following: immunomodulators, corticosteroids, or anti-TNF therapies
**Exclusion**
Gastrointestinal exclusion criteria<br>• Abdominal abscess, extensive colonic resection, subtotal or total colectomy<br>• History of >3 small bowel resections or diagnosis of short bowel syndrome<br>• Receipt of tube feeding, defined formula diets, or parenteral alimentation within 28 days before administration of the first dose of study drug<br>• Previous ileostomy, colostomy, or known fixed symptomatic stenosis of the intestine<br>• Receipt of any investigational or approved biologic/biosimilar within 60 days or 5 half-lives of screening, or receipt of any nonpermitted investigational or approved nonbiologic therapies within 30 days or 5 half-lives of screening<br>– Oral 5-ASA probiotics and antibiotics were permitted if doses were stable for 2 weeks prior to the first dose of the study and remained stable throughout the study. Antidiarrheals were permitted. Azathioprine, 6-mercaptopurine, or methotrexate could be continued if the patient's dose had been stable for 8 weeks prior to the start of the study

- Topical (rectal) treatment with 5-ASAs or corticosteroid enemas/suppositories within 2 weeks of administration of the first dose of study drug
- Requirement or anticipated requirement for surgical intervention for CD during the study
- History or evidence of adenomatous colonic polyps that had not been removed, or colonic mucosal dysplasia
- Suspected or confirmed diagnosis of ulcerative colitis, indeterminate colitis, ischemic colitis, radiation colitis, diverticular disease associated with colitis, or microscopic colitis

Infectious disease exclusion criteria

- Evidence of an active infection during the screening period
- Evidence of, or treatment for, *Clostridium difficile* infection or other intestinal pathogen within 28 days before the first dose of study drug
- Chronic hepatitis B (HBV) infection or chronic hepatitis C virus infection
  - Patients with HBV immunity may have been included
- Active or latent tuberculosis
- Any identified congenital or acquired immunodeficiency (eg, common variable immunodeficiency, HIV infection, organ transplantation)
- Receipt of any live vaccinations within 30 days prior to screening
- Clinically significant infection (eg, pneumonia, pyelonephritis) within 30 days prior to screening, or ongoing chronic infection

General exclusion criteria

- Previous exposure to approved or investigational anti-integrin antibodies (eg, natalizumab, efalizumab, etrolizumab, AMG 181), mucosal addressin cell adhesion molecule-1 (MAdCAM-1) antibodies or rituximab
- Previous exposure to vedolizumab
- Hypersensitivity or allergies to any vedolizumab excipients
- Any unstable or uncontrolled cardiovascular, pulmonary, hepatic, renal, gastrointestinal, genitourinary, hematological, coagulation, immunological, endocrine/metabolic, or other medical disorder that, in the opinion of the investigator, would confound the study results or compromise patient safety
- Any surgical procedure requiring general anesthesia within 30 days prior to screening or plans to undergo major surgery during the study period
- Any history of malignancy, except for the following: (a) adequately treated nonmetastatic basal cell skin cancer, (b) squamous cell skin cancer that had been adequately treated and that had not recurred for at least 1 year before screening, and (c) history of cervical carcinoma in situ that had been adequately treated and that had not recurred for at least 3 years before screening. Patients with remote history of malignancy (eg, >10 years since completion of curative therapy without recurrence) were to be considered on a case-by-case basis based on the nature of the malignancy and the therapy received
- History of any major neurological disorders, including stroke, multiple sclerosis, brain tumor, or neurodegenerative disease

- Positive progressive multifocal leukoencephalopathy subjective symptom checklist at screening (or before the administration of the first dose of study drug at week 0)
- Any of the following laboratory abnormalities during the screening period:
  - Hemoglobin level <8 g/dL
  - White blood cell count <3 × 10^9/L
  - Lymphocyte count <0.5 × 10^9/L
  - Platelet count <100 × 10^9/L or >1200 × 10^9/L
  - Alanine aminotransferase or aspartate aminotransferase >3 × upper limit of normal (ULN)
  - Alkaline phosphatase >3 × ULN
  - Serum creatinine >2 × ULN
- History of drug abuse (defined as any illicit drug use) or a history of alcohol abuse within 1 year before screening
- Active psychiatric problem that, in the investigator's opinion, may have interfered with compliance with study procedures
- Inability of patient or caregiver to attend all the study visits or comply with study procedures
- Unwillingness or inability to self-inject, or lack of a caregiver (defined as a legal adult) to inject the study medication
- Lactation or pregnancy during the screening period or a positive urine pregnancy test at week 0, before study drug administration
- Intention to reproduce before, during, or within 18 weeks after participating in this study
- Immediate family member, study site employee, or involvement in a dependent relationship with a study site employee who was involved in conduct of this study (eg, spouse, parent, child, sibling), or may have consented under duress

TABLE S3. Demographics and Baseline Characteristics for Patients With UC Who Transitioned From IV to SC

Characteristics	Transitioned to SC in OLE after VDZ infusions in VISIBLE 1 (n = 39)
Age, years, mean (SD)	41.3 (14.4)
Male, n (%)	23 (59.0)
Duration of UC, years, mean (SD)	9.1 (6.2)
Baseline disease activity, n (%)	
Moderate (Mayo score = 6 to 8)	13 (33.3)
Severe (Mayo score = 9 to 12)	26 (66.7)
Partial Mayo score <6	9 (23.1)
Partial Mayo score ≥6	30 (76.9)
Prior anti-TNF use, n (%)	17 (43.6)
Prior anti-TNF failure, n (%)[a]	17 (43.6)
Concomitant medications, n (%)[b]	
Corticosteroids	19 (48.7)
5-ASAs	34 (87.2)
Immunomodulators	11 (28.2)

Note: Baseline data are from baseline of VISIBLE 1 study. Thirty-nine patients enrolled in the VISIBLE OLE study after having IV infusions in VISIBLE 1. Not all received the full 8 doses of IV vedolizumab in VISIBLE 1.
[a]Based on total patients; not based on patients exposed to anti−TNF-α.
[b]Concomitant medications included those taken at any time during week 0 to week 52 of the VISIBLE 1 study.
Abbreviations: ASA, aminosalicylic acid; IV, intravenous; OLE, open-label extension; SC, subcutaneous; TNF, tumor necrosis factor; UC, ulcerative colitis; VDZ, vedolizumab.

TABLE S4. Demographics and Baseline Characteristics for Patients With UC Who Experienced Vedolizumab Treatment Interruptions in VISIBLE 1

	Placebo completers (46 weeks' treatment interruption) (n = 20)	Placebo early terminators (1−45 weeks' treatment interruption) (n = 32)	All patients with dose interruption (n = 52)
Age, y, mean (SD)	40.4 (9.76)	38.9 (13.16)	39.5 (11.88)
BMI, kg/m^2, mean (SD)	25.2 (5.13)	24.5 (6.57)	24.7 (6.01)
Male, n (%)	12 (60)	19 (59.4)	31 (59.6)
Smoking status, n (%)			
Never smoked	16 (80)	20 (62.5)	36 (69.2)
Ex-smoker	4 (20)	12 (37.5)	16 (30.8)
Current smoker	0	0	0
Duration of UC, y, mean (SD)	7.8 (7.09)	7.4 (7.48)	7.5 (7.26)
Disease localization, n (%)			
Extensive colitis	1 (5.0)	3 (9.4)	4 (7.7)
Left-sided colitis	11 (55.0)	12 (37.5)	23 (44.2)
Pancolitis	4 (20.0)	15 (46.9)	19 (36.5)
Proctosigmoiditis	4 (20.0)	2 (6.3)	6 (11.5)
Baseline disease activities, n (%)			
Total Mayo score			
Moderate (6 to 8)	6 (30.0)	12 (37.5)	18 (34.6)
Severe (9 to 12)	14 (70.0)	20 (62.5)	34 (65.4)
Partial Mayo score			
Moderate (<6)	5 (25.0)	8 (25.0)	13 (25.0)
Severe (≥6)	15 (75.0)	24 (75.0)	39 (75.0)
Stool frequency score, mean (SD)	2.4 (0.75)	2.3 (1.02)	2.3 (0.92)
Rectal bleeding score, mean (SD)	1.6 (0.69)	1.8 (0.77)	1.7 (0.74)
Fecal calprotectin, µg/g, mean (SD)	2216.1 (2388.4)	2613.0 (3278.7)	2460.3 (2949.1)
Prior TNF-α antagonist use failure, n (%)[a]	6 (30.0)	14 (43.8)	20 (38.5)
Concomitant corticosteroid use, n (%)	8 (40.0)	15 (46.9)	23 (44.2)
Concomitant immunomodulator use, n (%)	5 (25.0)	11 (34.4)	16 (30.8)

Note: Baseline data are from VISIBLE 1 baseline information.
[a]Based on total patients, not based on patients exposed to anti−TNF-α.
Abbreviations: BMI, body mass index; TNF, tumor necrosis factor, UC, ulcerative colitis; y, years.

TABLE S5. Demographics and Baseline Characteristics for Patients With CD Who Experienced Vedolizumab Dose Interruptions in VISIBLE 2

	Placebo completers (46 weeks treatment interruption) (n = 68)	Placebo early terminators (1-45 weeks' treatment interruption) (n = 46)	All patients with dose interruption (n = 114)
Age, y, mean (SD)	35.9 (12.46)	38.6 (13.99)	37.0 (13.11)
BMI, kg/m^2, mean (SD)	23.9 (5.94)	24.3 (5.26)	24.1 (5.65)
Male, n (%)	35 (51.5)	22 (47.8)	57 (50.0)
Smoking status, n (%)			
Never smoked	46 (67.6)	23 (50.0)	69 (60.5)
Ex-smoker	11 (16.2)	10 (21.7)	21 (18.4)
Current smoker	11 (16.2)	13 (28.3)	24 (21.1)
Prior surgery for CD, n (%)	24 (35.3)	9 (19.6)	33 (28.9)
Duration of CD, y, mean (SD)	7.8 (7.23)	9.8 (10.92)	8.6 (8.91)
Disease localization, n (%)			
Colon only	12 (17.6)	10 (21.7)	22 (19.3)
Ileum only	12 (17.6)	4 (8.7)	16 (14.0)
Ileocolonic	35 (51.5)	29 (63.0)	64 (56.1)
Other	9 (13.2)	3 (6.5)	12 (10.5)
History of fistulizing disease, n (%)	18 (26.5)	12 (26.1)	30 (26.3)
Fistula status, n (%)			
Draining	4 (5.9)	7 (15.2)	11 (9.6)
All closed	2 (2.9)	1 (2.2)	3 (2.6)
None	62 (91.2)	38 (82.6)	100 (87.7)
Baseline disease activity, n (%)			
Moderate (CDAI score ≤300)	35 (51.5)	31 (67.4)	66 (57.9)
Severe (CDAI score >300)	33 (48.5)	15 (32.6)	48 (42.1)
C-reactive protein, µg/g, mean (SD)	15.1 (23.33)	16.9 (20.48)	15.8 (22.15)
Fecal calprotectin, µg/g, mean (SD)	1374.8 (2249.13)	1631.0 (1733.88)	1476.8 (2055.01)
Prior TNF-α antagonist use failure, n (%)[a]	30 (44.1)	21 (45.7)	51 (44.7)
Concomitant corticosteroid use, n (%)	22 (32.4)	18 (39.1)	40 (35.1)
Concomitant immunomodulator use, n (%)	22 (32.4)	19 (41.3)	41 (36.0)

Note: Baseline data are from VISIBLE 2 baseline information.
[a]Based on total patients, not based on the patients exposed to anti−TNF-α.
Abbreviations: BMI, body mass index; CD, Crohn's disease; CDAI, Crohn's Disease Activity Index; TNF, tumor necrosis factor; y, years.

TABLE S6. AEs During Vedolizumab Treatment for UC Following Treatment Interruption in VISIBLE 1

	Placebo completers (46-week treatment interruption) (n = 20)		Placebo early terminators (1−45 weeks' treatment interruption) (n = 32)		All patients with dose interruption (n = 52)	
	Events n	Patients n (%)	Events n	Patients n (%)	Events n	Patients n (%)
Any AE	35	9 (45.0)	120	24 (75.0)	155	33 (63.5)
Related	6	2 (10.0)	50	8 (25.0)	56	10 (19.2)
Not related	29	7 (35.0)	70	16 (50.0)	99	23 (44.2)
Mild	28	5 (25.0)	95	9 (28.1)	123	14 (26.9)
Moderate	6	3 (15.0)	23	13 (40.6)	29	16 (30.8)
Severe	1	1 (5.0)	2	2 (6.3)	3	3 (5.8)
Serious AE	2	2 (10.0)	5	4 (12.5)	7	6 (11.5)
Not related	2	2 (10.0)	5	4 (12.5)	7	6 (11.5)
AEs leading to study drug discontinuation	1	1 (5.0)	1	1 (3.1)	2	2 (3.8)
Injection-site reactions	0	0	36	6 (18.8)	36	6 (11.5)
Hypersensitivity reactions[a]	1	1 (5.0)	5	2 (6.3)	6	3 (5.8)

Note: All AEs are treatment-emergent AEs (defined as an AE that starts or worsens on or after study day 1, which is the day of first treatment period dosing, and no later than the end of the extension period, which is the last dose from disposition-captured results + 126 days, or last contact date [for withdrawals], whichever is earliest).
[a]Hypersensitivity reactions include all events that are anaphylactic/anaphylactoid shock conditions SMQ (broad), angioedema SMQ (broad), or hypersensitivity SMQ (broad). "Broad" includes both broad and narrow selections.
Abbreviations: AEs, adverse events; SMQ, Standardized MedDRA [Medical Dictionary for Regulatory Activities] Queries; UC, ulcerative colitis.

TABLE S7. AEs During Vedolizumab Treatment for CD Following Treatment Interruption in VISIBLE 2

	Placebo completers (46-week treatment interruption) (n = 68)		Placebo early terminators (1−45 weeks' treatment interruption) (n = 46)		All patients with dose interruption (n = 114)	
	Events n	Patients n (%)	Events n	Patients n (%)	Events n	Patients n (%)
Any AE	138	45 (66.2)	174	35 (76.1)	312	80 (70.2)
Related	8	6 (8.8)	15	8 (17.4)	23	14 (12.3)
Not related	130	39 (57.4)	159	27 (58.7)	289	66 (57.9)
Mild	89	23 (33.8)	106	8 (17.4)	195	31 (27.2)
Moderate	43	17 (25.0)	60	21 (45.7)	103	38 (33.3)
Severe	6	5 (7.4)	8	6 (13.0)	14	11 (9.6)
Serious AE	9	8 (11.8)	16	11 (23.9)	25	19 (16.7)
Related	1	1 (1.5)	0	0	1	1 (0.9)
Not related	8	7 (10.3)	16	11 (23.9)	24	18 (15.8)
AEs leading to study drug discontinuation	2	2 (2.9)	3	2 (4.3)	5	4 (3.5)
Injection-site reactions	0	0	1	1 (2.2)	1	1 (0.9)
Hypersensitivity reactions[a]	1	1 (1.5)	7	5 (10.9)	8	6 (5.3)

Note: All AEs are treatment-emergent AEs (defined as an AE that starts or worsens on or after study day 1, which is the day of the first treatment period dosing, and no later than the end of the extension period, which is the last dose from disposition-captured results + 126 days, or the last contact date [for withdrawals], whichever is earliest).

[a]Hypersensitivity reactions include all events that are anaphylactic/anaphylactoid shock conditions SMQ (broad), angioedema SMQ (broad), or hypersensitivity SMQ (broad). "Broad" includes both broad and narrow selection.

Abbreviations: AEs, adverse events; CD, Crohn's disease; SMQ, Standardized MedDRA [Medical Dictionary for Regulatory Activities] Queries.

TABLE S8. Demographics and Baseline Characteristics for Patients With UC Who Received Vedolizumab Dose Escalations

	VISIBLE OLE Q2W/QW (n = 49)	VISIBLE 1 treatment failure (n = 21)	All patients with dose escalation (n = 70)
Age, y, mean (SD)	40.6 (13.13)	36.3 (13.77)	39.3 (13.37)
BMI, kg/m^2, mean (SD)	23.9 (5.79)	22.6 (3.78)	23.5 (5.27)
Male, n (%)	25 (51.0)	12 (57.1)	37 (52.9)
Smoking status, n (%)			
Never smoked	29 (59.2)	14 (66.7)	43 (61.4)
Ex-smoker	15 (30.6)	4 (19.0)	19 (27.1)
Current smoker	5 (10.2)	3 (14.3)	8 (11.4)
Duration of UC, y, mean (SD)	6.9 (6.1)	8.2 (7.36)	7.3 (6.48)
Disease localization, n (%)			
Extensive colitis	4 (8.2)	1 (4.8)	5 (7.1)
Left-sided colitis	19 (38.8)	10 (47.6)	29 (41.4)
Pancolitis	23 (46.9)	8 (38.1)	31 (44.3)
Proctosigmoiditis	3 (6.1)	2 (9.5)	5 (7.1)
Baseline disease activities, n (%)			
Total Mayo score			
Moderate (6 to 8)	10 (20.4)	7 (33.3)	17 (24.3)
Severe (9 to 12)	39 (79.6)	14 (66.67)	53 (75.7)
Partial Mayo score			
Moderate (<6)	8 (16.3)	7 (33.3)	15 (21.4)
Severe (≥6)	41 (83.7)	14 (66.7)	55 (78.6)
Stool frequency score, mean (SD)	2.6 (0.65)	2.3 (0.72)	2.5 (0.68)
Rectal bleeding score, mean (SD)	1.7 (0.79)	1.8 (0.89)	1.7 (0.82)
Fecal calprotectin, μg/g, mean (SD)	4127.1 (6857.58)	2931.4 (3265.67)	3768.4 (6009.15)
Prior TNF-α antagonist failure, n (%)[a]	26 (53.1)	6 (28.6)	32 (45.7)
Concomitant corticosteroid use, n (%)	26 (53.1)	16 (76.2)	42 (60.0)
Concomitant immunomodulator use, n (%)	19 (38.8)	8 (38.1)	27 (38.6)

Note: Baseline data are from VISIBLE 1 baseline information.
[a]Based on total patients; not based on the patients exposed to anti−TNF-α.
Abbreviations: BMI, body mass index; OLE, open-label extension; Q2W, every 2 weeks; QW, once per week; TNF, tumor necrosis factor; UC, ulcerative colitis; y, years.

TABLE S9. Demographics and Baseline Characteristics for Patients With CD Who Received Vedolizumab Dose Escalations

	VISIBLE OLE Q2W/QW (n = 63)	VISIBLE 2 treatment failure (n = 68)	All patients with dose escalation (n = 131)
Age, y, mean (SD)	37.6 (13.69)	37.3 (14.05)	37.4 (13.83)
BMI, kg/m^2, mean (SD)	23.9 (5.89)	25.0 (6.52)	24.5 (6.23)
Male, n (%)	35 (55.6)	32 (47.1)	67 (51.1)
Smoking status, n (%)			
Never smoked	34 (54.0)	44 (64.7)	78 (59.5)
Ex-smoker	16 (25.4)	16 (23.5)	32 (24.4)
Current smoker	13 (20.6)	8 (11.8)	21 (16.0)
Prior surgery for CD, n (%)	21 (33.3)	20 (29.4)	41 (31.3)
Duration of CD, y, mean (SD)	8.9 (7.18)	9.8 (7.94)	9.3 (7.57)
Disease localization, n (%)			
Colon only	13 (20.6)	16 (23.5)	29 (22.1)
Ileum only	9 (14.3)	19 (27.9)	28 (21.4)
Ileocolonic	32 (50.8)	24 (35.3)	56 (42.7)
Other	9 (14.3)	9 (13.2)	18 (13.7)
History of fistulizing disease, n (%)	15 (23.8)	17 (25.0)	32 (24.4)
Fistula status, n (%)			
Draining	3 (4.8)	5 (7.4)	8 (6.1)
All closed	3 (4.8)	4 (5.9)	7 (5.3)
None	57 (90.5)	59 (86.8)	116 (88.5)
Baseline disease activity, n (%)			
Moderate (CDAI score ≤300)	41 (65.1)	33 (48.5)	74 (56.5)
Severe (CDAI score >300)	22 (34.9)	35 (51.5)	57 (43.5)
C-reactive protein, µg/g, mean (SD)	13.8 (19.79)	20.6 (33.68)	17.4 (27.99)
Fecal calprotectin, µg/g, mean (SD)	1480.2 (2170.77)	1588.7 (1860.76)	1536.6 (2008.68)
Prior TNF-α antagonist use failure, n (%)[a]	38 (60.3)	45 (66.2)	83 (63.4)
Concomitant corticosteroid use, n (%)	24 (38.1)	27 (39.7)	51 (38.9)
Concomitant immunomodulatory, use n (%)	19 (30.2)	21 (30.9)	40 (30.5)

Note: Baseline data are from VISIBLE 2 baseline information.
[a]Based on total patients, not based on the patients exposed to anti−TNF-α.
Abbreviations: BMI, body mass index; CD, Crohn's disease; CDAI, Crohn's Disease Activity Index; OLE, open-label extension; Q2W, every 2 weeks; QW, once per week; TNF, tumor necrosis factor; y, years.

TABLE S10. AEs Following Vedolizumab Dose Escalation in Patients With UC

	Week 14 responders and VISIBLE 1 completers VISIBLE OLE Q2W/QW (n = 49)		VISIBLE 1 treatment failure (n = 21)		All patients with dose escalation (n = 70)	
	Events n	Patients n (%)	Events n	Patients n (%)	Events n	Patients n (%)
Any AE	122	34 (69.4)	64	14 (6.7)	186	48 (68.6)
Related	17	9 (18.4)	10	6 (28.6)	27	15 (21.4)
Not related	105	25 (51.0)	54	8 (38.1)	159	33 (47.1)
Mild	85	13 (26.5)	32	3 (14.3)	117	16 (22.9)
Moderate	29	14 (28.6)	30	9 (42.9)	59	23 (32.9)
Severe	8	7 (14.3)	2	2 (9.5)	10	9 (12.9)
Serious AE	16	13 (26.5)	7	4 (19.0)	23	17 (24.3)
Related	2	1 (2.0)	1	1 (4.8)	3	2 (2.9)
Not related	14	12 (24.5)	6	3 (14.3)	20	15 (21.4)
AEs leading to study drug discontinuation	5	5 (10.2)	1	1 (4.8)	6	6 (8.6)

Note: All AEs are treatment-emergent AEs that started after escalating to QW. A treatment-emergent AE was defined as an AE that starts or worsens on or after study day 1, which is the day of the first treatment period dosing, and no later than the end of the extension period, which is the last dose from disposition-captured results + 126 days or the last contact date (for withdrawals), whichever is earliest. Abbreviations: AEs, adverse events; OLE, open-label extension; Q2W, every 2 weeks; QW, once per week; UC, ulcerative colitis.

TABLE S11. AEs Following Vedolizumab Dose Escalation in Patients With CD

	Week 14 responders and VISIBLE 2 completers VISIBLE OLE Q2W/QW (n = 63)		VISIBLE 2 treatment failure (n = 68)		All patients with dose escalation (n = 131)	
	Events n	Patients n (%)	Events n	Patients n (%)	Events n	Patients n (%)
Any AE	95	34 (54.0)	218	57 (83.8)	313	91 (69.5)
Related	10	5 (7.9)	38	20 (29.4)	48	25 (19.1)
Not related	85	29 (46.0)	180	37 (54.4)	265	66 (50.4)
Mild	61	18 (28.6)	130	16 (23.5)	191	34 (26.0)
Moderate	29	12 (19.0)	73	31 (45.6)	102	43 (32.8)
Severe	5	4 (6.3)	15	10 (14.7)	20	14 (10.7)
Serious AE	10	9 (14.3)	22	17 (25.0)	32	26 (19.8)
Related	1	1 (1.6)	2	2 (2.9)	3	3 (2.3)
Not related	9	8 (12.7)	20	15 (22.1)	29	23 (17.6)
AEs leading to study drug discontinuation	2	2 (3.2)	3	2 (2.9)	5	4 (3.1)

Note: All AEs are treatment-emergent AEs that started after escalating to QW. A treatment-emergent AE was defined as an AE that starts or worsens on or after study day 1 (defined as the day of the first treatment period dosing), and no later than the end of the extension period (defined as the last dose from disposition-captured results + 126 days or the last contact date [for withdrawals], whichever is earliest).

Abbreviations: AEs, adverse events; CD, Crohn's disease; OLE, open-label extension; Q2W, every 2 weeks; QW, once per week.

Figure S1. (A) VISIBLE 1 study design[1]; (B) VISIBLE 2 study design.[2]
CD, Crohn's disease; CS, corticosteroid; IMMs, immunomodulators; IV, intravenous; Q2W, every 2 weeks; Q8W, every 8 weeks; SC, subcutaneous; TNF, tumor necrosis factor; UC, ulcerative colitis.

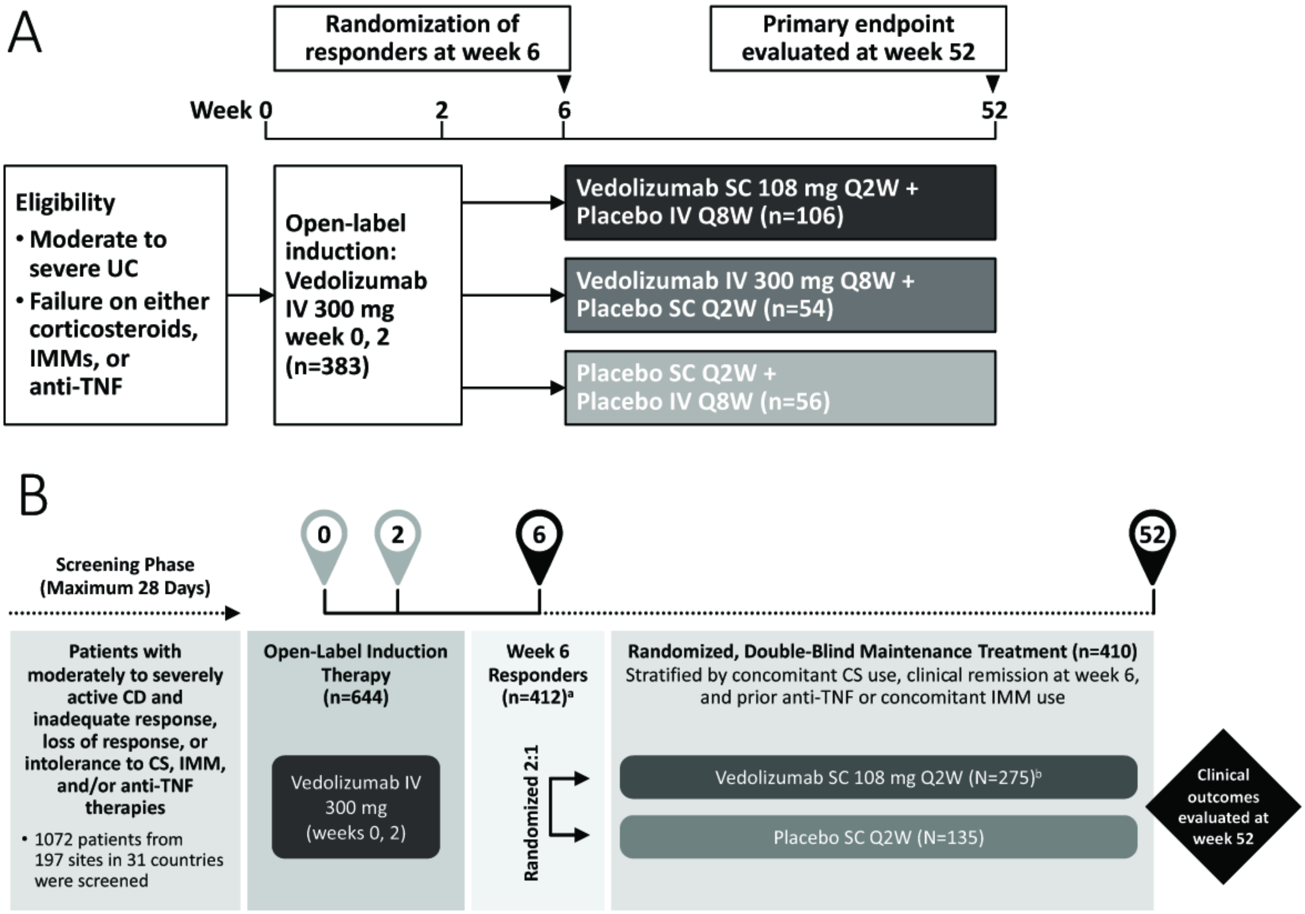

Figure S2. Patient disposition and patient populations in the 4 post hoc analyses. CD, Crohn's disease; IV, intravenous; OLE, open-label extension; PBO, placebo; Q2W, every 2 weeks; QW, once every week; SC, subcutaneous; UC, ulcerative colitis; VDZ, vedolizumab.

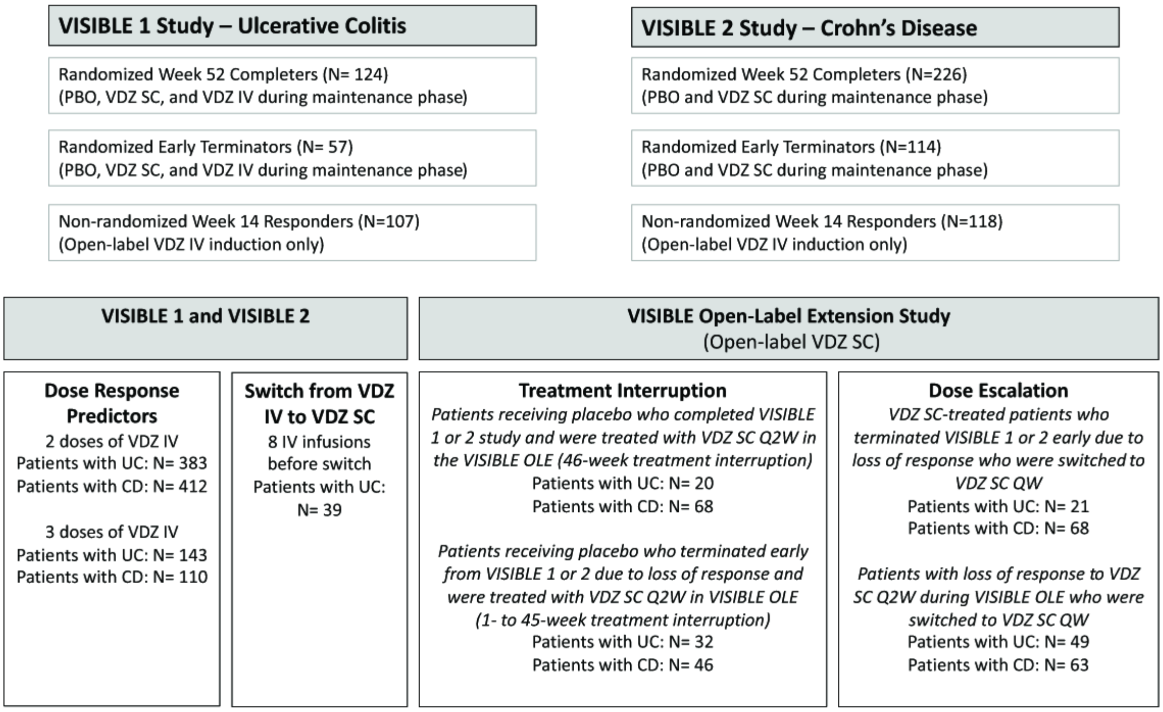

References

1. Sandborn WJ, Baert F, Danese S, et al. Efficacy and safety of vedolizumab subcutaneous formulation in a randomized trial of patients with ulcerative colitis. *Gastroenterology.* 2020;158(3):562−572.e512.
2. Vermeire S, D'Haens G, Baert F, et al. Efficacy and safety of subcutaneous vedolizumab in patients with moderately to severely active Crohn's disease: results from the VISIBLE 2 randomized trial. *J Crohns Colitis.* 2022;16(1):27−38.
